# Supplementary material for: Valproic Acid Improves Antisense-Mediated Exon-Skipping Efficacy in mdx Mice
Source: Int J Mol Sci. 2025 Mar 13;26(6):2583. doi: 10.3390/ijms26062583 (PMC11942597; doi:10.3390/ijms26062583)
Supplement: Supplementary file 1 [file ijms-26-02583-s001.zip › ijms-3451166-supplementary.pdf]

Supplementary material

Valproic Acid Improves Antisense-Mediated Exon-Skipping Efficacy in *mdx* Mice

Micky Phongsavanh [1,†], Flavien Bizot [1,†], Amel Saoudi [1], Cecile Gastaldi [2,3], Olivier Le Coz [1], Thomas Tensorer [4], Elise Brisebard [5], Luis Garcia [1,2] and Aurélie Goyenvalle [1,2,*]

[1] Université Paris-Saclay, UVSQ, Inserm, END-ICAP, 78000 Versailles, France; xaysongkhame-micky.phongsavanh@uvsq.fr (M.P.); olivier.le-coz@uvsq.fr (O.L.C.); luis.garcia@uvsq.fr (L.G.)
[2] Medical Biology Department, Centre Scientifique de Monaco, 98000 Monaco, Monaco; cgastaldi@centrescientifique.mc
[3] LIA BAHN, CSM-UVSQ, 98000 Monaco, Monaco
[4] SQY Therapeutics, UVSQ, 78180 Montigny le Bretonneux, France
[5] INRAE Oniris, UMR 703 PAnTher, 44300 Nantes, France
* Correspondence: aurelie.goyenvalle@uvsq.fr
† These authors contributed equally to the work.

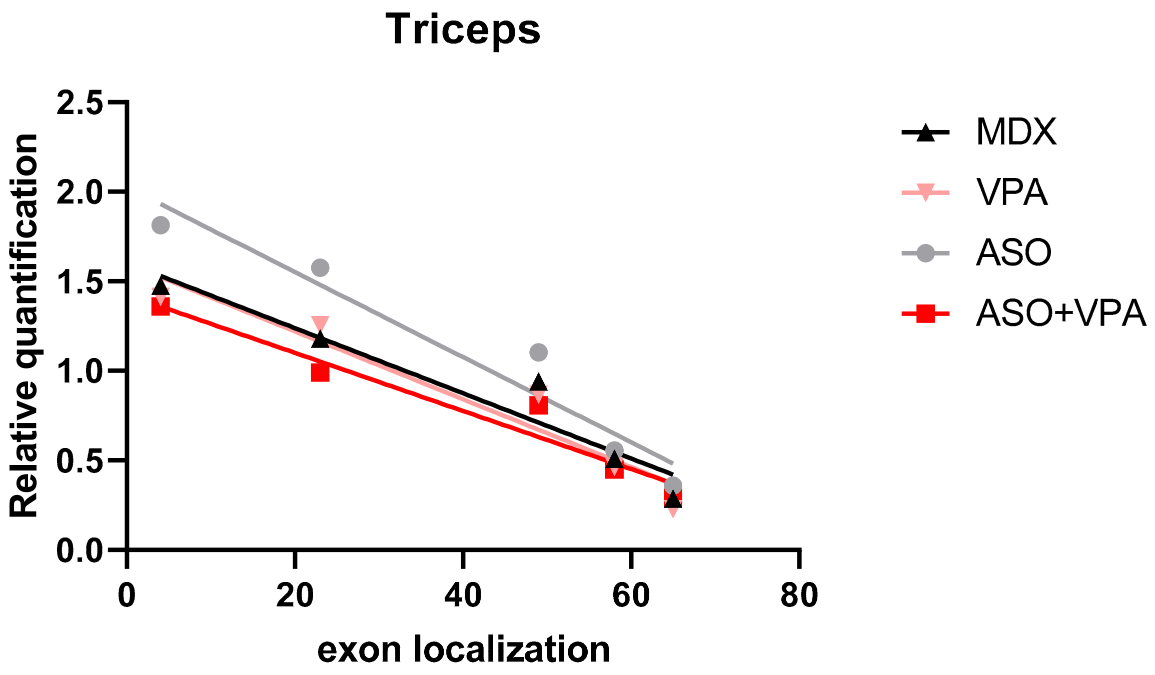

Figure S1. Relative expression of 5′-3′ *Dmd* transcript imbalance in triceps analyzed by taqman qPCR at different exon junctions. Results are expressed as mean ± SEM; n=6-8 mice per group.

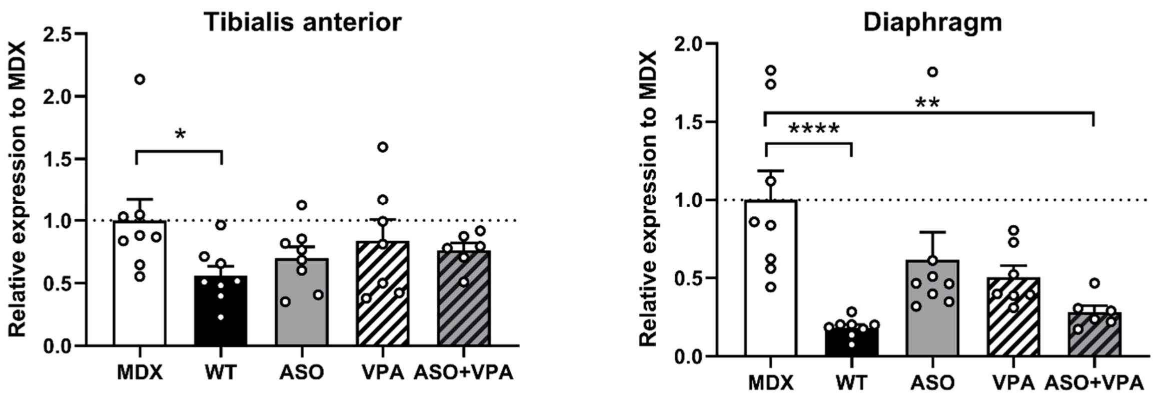

Figure S2. Relative quantification of follistatin expression in tibialis anterior and diaphragm by qPCR. Results are expressed as mean ± SEM; n=6-8 mice per group. * $p < 0.05$, ** $p < 0.01$, and **** $p < 0.0001$ compared to *mdx* saline, analyzed by one-way ANOVA.

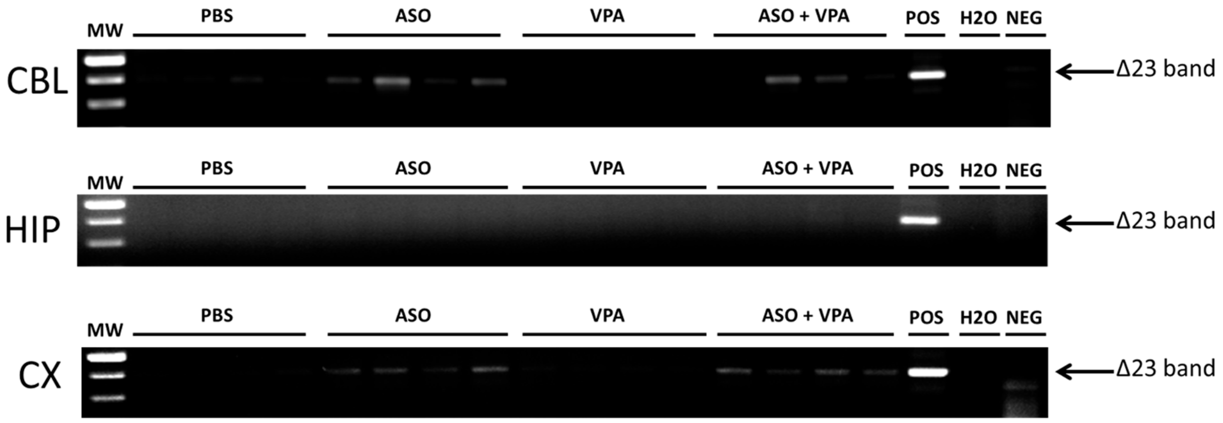

Figure S3. Evaluation of exon 23 skipping using RT-PCR with a specific primer across the skipped junction 22-24 performed on brain tissues (cerebellum, hippocampus, cortex) of *mdx* mice treated with PBS, ASO, VPA, or the ASO+VPA combination. N=4 mice per group. A band corresponding to the exon 23 skipped product is clearly detected in the positive control sample (POS) corresponding to a muscle tissue (TA) where exon skipping was previously confirmed. A faint band corresponding to exon 23 skipped product is observed in cerebellum and cortex samples from mice treated with ASO or ASO+VPA, but not in PBS or VPA treated samples.
